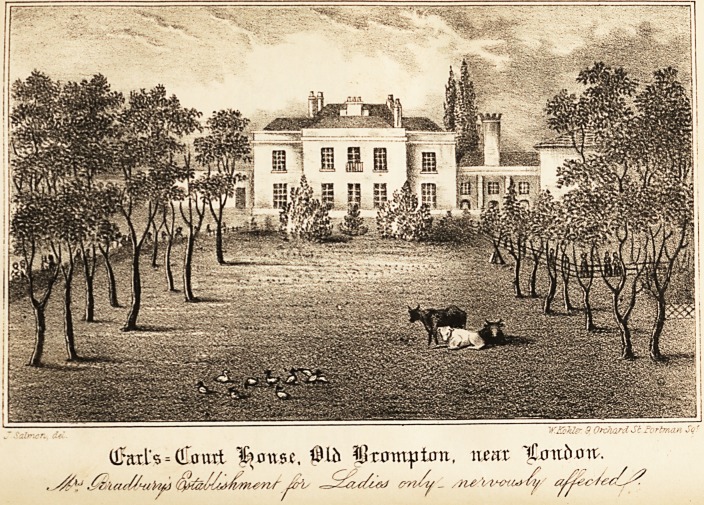# Earl's-Court House, Old Brompton, near London. Mrs. Bradbury's Establishment for the Care and Recovery of Ladies Labouring under Nervous Affection

**Published:** 1850-10-01

**Authors:** 


### EARL'S-COURT HOUSE, OLD BROMPTON,

### NEAR LONDON.

### MRS. BRADBURY'S ESTABLISHMENT

### FOR THE CARE AND RECOVERY OF LADIES LABOURING UNDER NERVOUS AFFECTION.

---

"Endeavour to be first in thy calling, whatever it be; neither let any one go before thee in well-doing; nevertheless, do not envy the merits of another, but improve thine own talents."—ECONOMY OF HUMAN LIFE.

---

MRS. BRADBURY, assisted by a lady in whom she has the most implicit confidence, receives a limited number of LADIES, labouring under nervous complaints, at her Establishment, Earl's-court House, Old Brompton, two miles from Hyde-park Corner.

Testimonials are subjoined from gentlemen eminent in the medical profession, as well as from persons of consideration, in commendation of this Establishment. It has been regarded by them as affording an eligible retreat, and as being in every way adapted to all conditions and circumstances of mental malady,—either where a public asylum was not required, or the connexions of the patient considered it unsuitable.

The *permanent cure* of the disorder is the paramount object in view. However long it has existed, in the moral and medical treatment the hope of recovery is never lost sight of; whilst, at the same time, every attention to the feelings and inclination of the patients (as far as is consistent with propriety) is always observed. There is no deviation from the general outline but such as may be observed in the regulation of a large and well-conducted private family.

Religious consolation is regarded as an object of primary importance; not only are prayers, with a portion of Scripture, read every morning, but the constant attendance of a clergyman of the established church is afforded, who also performs weekly service in the establishment, while an opportunity of attending public worship is always given to those ladies capable of deriving benefit from its advantages; (such also are permitted to be visited by clergymen of their own persuasion at the request of their friends.)

Many are the instances wherein the cultivation, or rather the re-development, of original acquirements in literature and the fine arts has proved serviceable: this conviction induces Mrs. Bradbury to encourage by every possible means a disposition to renew such attainments.

The medical superintendence is effected by a physician of the first eminence in nervous diseases, and also by a gentleman in extensive practice, a member of the Royal College of Surgeons, who lay claim to a sound, rational, and effective mode of treatment of such affections in general; the basis consisting in the temporary separation from friends and connexions, the absence of all coercion, wholesome occupation agreeable to the individual's former moral habits and inclinations, and the application of remedial means calculated to allay nervous disturbance.

It must be obvious, that cases of recovery in this establishment, though most interesting, do not, from motives of delicacy and confidence, admit of publicity; but undeniable testimony can be adduced of many who have thus been restored to the perfect possession of mental enjoyment.

Professional gentlemen attending their own patients, may confidently rely that their views and directions will be implicitly adopted, and that there will be no interference whatever in their medical treatment.

In the construction of the establishment, every suggestion has been attended to that could minister to the comfort, amusement, and healthful gratification of inmates so imperatively demanding every solicitude and sympathy. Spacious corridors have been erected, warmed, and ventilated, upon the most improved principles, for the purpose of exercise in damp or inclement weather, where self-acting musical instruments, with various devices and means of diversion, are placed; whilst in the garden and grounds are swings, Merlin and easy chairs, for pleasure and recreation. A billiard-room and library are attached, with periodicals and other publications, and every possible means adopted that tends to draw the mind from its state of aberration, and thus progressively prepare it for a return to home and society.

The house is replete with every domestic convenience, is fitted up with hot, cold, shower, vapour, and medicated baths, and is of such magnitude as to admit of being divided into different compartments for the purpose of complete separation and classification, often found in such cases so highly desirable. It is situate on a gravelly soil, surrounded by extensive gardens and pleasure grounds, in which a farm and cows are included, combining all the advantages of rural cheerfulness with quietude and repose.

It was long the favoured residence of the celebrated *John Hunter*, and is considered by the faculty, from the salubrity of its temperature, the excellence of its springs, with many other advantages, to be the Montpelier of the metropolis.

*Distinct sitting rooms may be had, with the invalid's servants, if necessary, with the use of a carriage; and persons carefully selected for their humanity and experience are always in readiness to attend ladies at their own residences.*

The TERMS are varied according to the accommodation and attendance required, and although embracing the most liberal scale, *will not be found beyond the means of a moderate income.*

### TESTIMONIALS.

---

"Dec. 16, 1834.

"Having visited Mrs. Bradbury's house, at Earl's-court, with great attention, several times, and examined the new arrangements made in it for the accommodation of its inmates, in which their comfort and amusement are so much considered, I do not hesitate to pronounce it the very best establishment for the reception of insane patients which I have ever seen.

"HENRY HALFORD,

"President of the College of Physicians."

---

"Dec. 24, 1834.

"Having repeatedly had occasion to visit Mrs. Bradbury's establishment, Earl's-court House, I can very confidently state that, in convenience of accommodation, judicious arrangement, kind and gentle treatment, and the most ingenious and complete dispositions for affording perpetual amusement and occupation of mind for the afflicted, it comprehends greater capabilities for the restoration of health, and is therefore far superior to those advantages that can be obtained in any private abode whatever.

"JOHN HOWSHIP,

"Member of the Council of the Royal College of Surgeons, London.

"Saville Row."

"Dec. 29, 1834.

"The undersigned has had occasion, not unfrequently, to visit Earl's-court House, and he had an opportunity very lately of viewing attentively the additions and improvements which have been made there. He considers it but justice to state, that he has never seen any establishment (of a similar nature) in which more numerous or more excellent arrangements exist for administering to the health, the comfort, and the amusement of the patients: and, in his opinion, too much praise cannot be bestowed on Mrs, Bradbury for making such pecuniary sacrifices to render the house, with its offices and surrounding grounds, so desirable for the reception of ladies affected with the various forms of mental disease.

"W. G. MATON, M.D.

"Physician to H.R.H. the Duchess of Kent,
and to the Princess Victoria.

"New Street, Spring Gardens."

---

"Jan. 10, 1835.

"I have visited with attention Mrs. Bradbury's house at Earl's-court, both before and since the recent alterations and improvements which have been made there, and I have no hesitation in stating it as my opinion, that it is an establishment of a very superior order for the reception of insane ladies, containing, as it does, not only all the requisite accommodation for their management and security, but being replete also within itself and its surrounding grounds with every comfort and luxury. I may add, that it is calculated to secure to the patient, should it be required, the seclusion and quiet of a private house or lodging, in conjunction with all the advantages of a large, airy, and commodious mansion.

"W. F. CHAMBERS, M.D.

"Physician to St. George's Hospital.

"Lower Brook Street, Bond Street."

---

"Feb. 28, 1835.

"I have, with great attention and minuteness, examined Mrs. Bradbury's establishment, and highly approve of its numerous and judicious arrangements. It affords all the comforts and best means of occupying the minds of those afflicted with the various forms of mental imbecility. It contains all the requisite accommodation for the moral and medical treatment of insane persons. It is, in my opinion, an asylum of a very superior description, and the best calculated I have ever seen for the treatment and cure of insane persons. The greatest praise is due to Mrs. Bradbury for having expended a large amount of capital on this complete and unequalled establishment, and for having devoted it to ladies exclusively. I can also conscientiously add, that the kind and gentle treatment invariably practised by that lady to her unfortunate inmates is entitled to the highest commendation, which, with the advantages of her judicious system of treatment, tend to the restoration of health better than any private abode, however excellent.

"M. RYAN, M.D.

"Editor of the London Medical and Surgical Journal.

"Great Queen Street,
St. James's Park."

"15, Welbeck Street, April 20, 1835.

"Mrs. Bradbury's present arrangements of her establishment for insane ladies may be considered as complete. All that vigilant superintendence, without irksome or unnecessary restraint—all that quietude and tranquillity, without gloom—all that rational amusement and diversion, without mental fatigue—can achieve in the restoration of persons thus unhappily situated, is here combined.

"By a judicious division of the premises (which will be viewed with interest by the medical practitioner, as having been the residence of the celebrated John Hunter,) an advantageous classification of the patients is effected;—free exercise can be enjoyed without the gaze of curiosity, and without the belief even being excited in the mind of the convalescent patient that her malady is known to any one beyond her own immediate attendant. Mrs. Bradbury is deserving of every encouragement for employing the best means for mitigating so severe a calamity, and for restoring those to health who may require for a time a separation from their families and their affairs.

"G. L. ROUPELL, M.D.

"Physician to St. Bartholomew's Hospital."

---

"Nov. 24, 1835.

"This establishment of Mrs. Bradbury, at Earl's-court, is remarkably well suited, in the size and healthy situation of the house, the arrangement of the apartments, the ample extent and disposition of the surrounding grounds, for the reception and care of deranged persons, and for affording them every opportunity of exercise, occupation, and amusement. These important advantages are rendered available in their full extent to the recovery, the safe custody, and the comfortable residence of the inmates, according to their various cases, by the amiable and kind feelings, the humane and judicious management, of Mrs. Bradbury, which I have had repeated opportunities of observing.

"Wm. LAWRENCE, F.R.S.

"Whitehall Place."

---

"Nov. 30, 1835.

"I have recently had frequent opportunities of observing the general management of Mrs. Bradbury's establishment at Earl's-court, into which females only are received, and in my opinion, it is admirably adapted as an asylum for nervous patients. The house is spacious, and the domestic arrangements comprise everything calculated to promote the comfort, the security, and recovery of the insane. The grounds are extensive and well-arranged; and when the symptoms of the disorder admit of amusements, the variety and selection appear to be well suited to the purpose. I can testify with confidence the kindness of Mrs. Bradbury and her assistants, and that the patients in this establishment are treated with the greatest judgment and humanity.

"A. TWEEDIE, M.D.

"Montague Place, Bedford Square."

---

"19, St. Mary Abbot's Terrace,
"Kensington, July 2, 1841.

"I have frequently visited Mrs. Bradbury's establishment, at Old Brompton, and have lately had the opportunity of minutely examining, and, thereby, becoming ultimately acquainted with all its internal arrangements. Of the whole, I have much pleasure in speaking in terms of the highest commendation.

" The establishment is exceedingly well adapted to promote the comfort, security, and recreation of patients labouring under every form of mental aberration, and the very great liberality displayed by Mrs. Bradbury, for the promotion of these objects, offers greater advantages to patients thus afflicted, than any other asylum of the kind with which I am acquainted. I may add, most truly and conscientiously, that the very kind and judicious treatment ever evinced by Mrs. Bradbury and her assistants towards her afflicted inmates, is creditable to them in the highest degree, and renders the establishment peculiarly desirable for the reception of patients labouring under every form of mental disease.

"HEATHFIELD TUPPER,
" Member of the Royal College of Surgeons, and
Surgeon to the Kensington Dispensary."

---

" If in the moral treatment and custody of the melancholic and insane, kindness combined with firmness, occupation of the various faculties, intellectual as well as physical, without satiety; and perfect security without the impression of restraint, be desirable requisites; these will be found in the highest perfection, in the establishment at Earl's-court House, conducted, and presided over, by Mrs. Bradbury.

" It is due to this lady, to promulgate the fact, that in addition to the refinement and appliances calculated to soothe, to engage, and to amuse the more tractable of her inmates, she, more than twenty years ago, adopted and fully carried out in her establishment, the system of conciliation, and non-restraint, now deemed so essential and salutary towards the more boisterous. This suggestion, as well as the discreet administration of the principle, was the offspring of her own superior intellect, the result of which, has been great success in the restoration of persons submitted to her charge. This power is equally displayed in the management of her whole establishment, (in which she is now assisted by an energetic and experienced coadjutor) which exhibits all the characteristics of a well conducted family, or a boarding house, rather than an asylum for the invalid.

" JOSEPH MOORE, M.D.

" Saville Row,
" Dec. 1842."

---

" Having inspected Earl's-court House, I have much pleasure in saying, that the mild and liberal treatment, and excellent classification of the patients, as well as the general good management observed there, reflects the highest credit upon Mrs. Bradbury, and, in my opinion, entitles her establishment to be considered one of the best in this country, for the security and comfort of ladies afflicted with nervous maladies.

" R. J. HOOPER, Surgeon.

" London Road, Southwark,
" Feb. 11, 1843."

"I have had occasion repeatedly to visit Mrs. Bradbury's establishment for insane ladies, at Earl's-court House, Old Brompton, and have great pleasure in expressing my satisfaction with all the arrangements, for the accommodation and management of the inmates.

"ROBERT LISTON,

" 5, Clifford Street,
" March 25, 1843."

---

" During the absence of the regular medical attendant, I professionally visited Earl's-court House, and thus had ample opportunity of judging of its superior merits as a retreat for the nervously affected. It is carried on with the greatest liberality and solicitude, the mild and affectionate system adopted by Mrs. Bradbury has been most successful. The number of cures effected, speak more in its favour than any individual testimony; yet I cannot refrain from expressing my opinion, that it is invaluable, and congratulate the friends of the mentally infirm that such an establishment exists.

" MATTHEW BAGNELL LEFEBURE,
" Member of the Royal College
of Surgeons, London.

" Castle Town,
Berehaven,
Ireland,
" April 18, 1843."

---

" Having repeatedly had occasion to visit Mrs. Bradbury's establishment at Earl's-court, and being acquainted with that lady, and her method of treating those mentally afflicted, I can conscientiously recommend her system, under which patients enjoy as much liberty as is consistent with their safety, and are rather watched and restrained than coerced. Her benevolence of character and long experience in the management of these cases, has taught her, regardless of trouble and expense, to apply in the most liberal manner every invention that has yet been devised to amuse and instruct the patients. By the constant vigilance and perpetual supervision of Mrs. Bradbury and her assistants, the utmost caution is exercised that the plans of treatment may be carried out by the attendants with proper gentleness and respect, and that no undue harshness shall ever be permitted. It affords me great satisfaction to be able also to testify to the obvious salubrity of the situation, and to the unusual extent and beauty of the pleasure grounds. Altogether, the arrangements are eminently calculated to promote the restoration of mental and physical health.

"H. P. ROBARTS,

"Fellow of the Royal College of Surgeons.

"11, Great Coram Street,
"4th July, 1848,"

---

"27th July, 1848.

"Having had frequent occasion to visit this establishment, 'so long and highly reputed for the cure and relief of those suffering from mental disease,' I can with much truth accord to its management, under Mrs. Bradbury, a constant, untiring energy and devotion towards the care, comfort, and even amusement of the patients. Earl's-court

House is large and well ordered, with a wide range of pleasure ground, and a cottage detached.

"The institution is quiet and retired, although so near the metropolis, and was formerly the residence of the celebrated surgeon and comparative anatomist, John Hunter.

"W. TURNER, M.R.S., L.A.C.

"31, Lower Phillimore Place, Kensington."

---

"I have frequently visited Earl's-court House, and have had opportunities of examining the arrangements and mode of conducting Mrs. Bradbury's establishment, and I highly approve of the kind and judicious method of treatment, as I have seen the salutary effects of this management very strikingly exemplified in one case under my care.

"I would strongly recommend this retreat to the notice of all parties desirous of securing the advantages of kind and conciliatory attention to the insane. The grounds are spacious and airy, and the house is in every respect suitable for the reception of ladies nervously affected.

"JAMES C. CUMMING, M.D.

"Lowndes Street, Belgrave Square,
"1st October, 1848."

---

"I have great pleasure in affording my testimony to the kindness and order with which the establishment at Earl's-court House, under the care of Mrs. Bradbury, is conducted. Not only is everything done for the peculiar class of cases which are treated under its roof, which medical science or humanity can suggest, but the situation of the place itself is one peculiarly well adapted for assisting the judicious efforts of Mrs. Bradbury and her coadjutors. I have personally witnessed the arrangements at Earl's-court House, and can conscientiously state that they meet with my cordial approval, as being well adapted to promote a restoration to health, where such an event is possible; and where it is not, to secure the greatest amount of comfort and enjoyment compatible with the condition of its afflicted inmates.

"EDWIN LANKESTER, M.D., F.R.S.,
" Physician to the Royal Verulam Dispensary.

" 22, Old Burlington Street,
" 12th October, 1848."

---

" From a knowledge of Earl's-court House during several years, I can bear testimony to its excellent management under the very judicious superintendence of Mrs. Bradbury, and to her admirable arrangements for the care, kind treatment, and recovery of the insane ladies under her charge. The house is spacious, airy, well situated, and excellently adapted for these important objects. The grounds are extensive and well laid out, combining ample accommodation for the recreation and exercise of the patients, with perfect security for their safety and retirement.

"R. I. POLLOCK, Surgeon.

" Path Place, Kensington,
"Oct. 13, 1848."

"Having some time since had the medical superintendance of Earl's-court House, conducted by Mrs. Bradbury, I was enabled to observe closely the internal arrangements, as well as the judicious classification adopted in this important establishment.

"Nothing can, in my opinion, contribute more to the restoration of a disordered mind, than the peculiar salubrity of the air, extent of pleasure grounds, most admirably arranged, as well for the amusement as the security of the unfortunate inmates, which this retreat affords in an eminent degree. To these advantages are added the long and extensive experience, humane and zealous treatment of Mrs. Bradbury, and her able coadjutor, which cannot fail to render it one of the most desirable asylums in the neighbourhood of London.

"JOHN PROPERT.

"New Cavendish Street,
"Oct. 14, 1848."

Savill & Edwards, Printers, 4, Chandos Street, Covent Garden.

## Figures and Tables

**Figure f1:**